# Predicting temporal variation in zooplankton beta diversity is challenging

**DOI:** 10.1371/journal.pone.0187499

**Published:** 2017-11-02

**Authors:** Vanessa Guimarães Lopes, Christina W. Castelo Branco, Betina Kozlowsky-Suzuki, Izidro F. Sousa-Filho, Leonardo Coimbra e Souza, Luis Mauricio Bini

**Affiliations:** 1 Departamento de Ecologia, Universidade Federal de Goiás, Goiás, Brazil; 2 Departamento de Zoologia, Universidade Federal do Estado do Rio de Janeiro, Rio de Janeiro, Brazil; 3 Departamento de Ecologia e Recursos Marinhos, Universidade Federal do Estado do Rio de Janeiro, Rio de Janeiro, Brazil; 4 Laboratório de Radioisótopos Eduardo Penna Franca (IBCCF/CCS), Universidade Federal do Rio de Janeiro, Rio de Janeiro, Brazil; Stockholm University, SWEDEN

## Abstract

Beta diversity, the spatial variation in species composition, has been related to different explanatory variables, including environmental heterogeneity, productivity and connectivity. Using a long-term time series of zooplankton data collected over 62 months in a tropical reservoir (Ribeirão das Lajes Reservoir, Rio de Janeiro State, Brazil), we tested whether beta diversity (as measured across six sites distributed along the main axis of the reservoir) was correlated with environmental heterogeneity (spatial environmental variation in a given month), chlorophyll-*a* concentration (a surrogate for productivity) and water level. We did not found evidence for the role of these predictors, suggesting the need to reevaluate predictions or at least to search for better surrogates of the processes that hypothetically control beta diversity variation. However, beta diversity declined over time, which is consistent with the process of biotic homogenization, a worldwide cause of concern.

## Introduction

The reasons why biodiversity varies spatially and temporally have always intrigued community ecologists. Both theoretical and practical issues have motivated the search for the underlying mechanisms of these variations. From a theoretical point of view, "what determines species diversity?" has been considered one of the most challenging questions in ecology [1, 2]. From a practical perspective, the relationship between biodiversity and ecosystem functioning (e.g., nutrient cycling, pollination, biomass production, water purification and invasive species resistance), with direct implications for human well-being, has been convincingly demonstrated by systematic reviews [3,4].

According to a search in the Web of Science database in July 03, 2017, the number of records with the terms (biodiversity AND spatial) and (biodiversity AND temporal), both in the field TOPIC and refined by the research area “Environmental Sciences Ecology” was about 8474 and 2830, respectively. Thus, spatial analyses of biodiversity are much more frequent than temporal analysis [5]. Temporal analyses of beta diversity (changes in species composition among local communities in a given area) are even rarer. For example, the numbers of records using the search parameters mentioned above and only changing the word "biodiversity" to "beta diversity" were equal to 1136 and 263, respectively. The paucity of temporal analyses of beta diversity can be explained by the difficulty in obtaining species composition data in different locations over time. In practical terms, the scarcity of compositional data at spatial and temporal scales greatly limits our understanding and capability of suggesting solutions for the fast-growing problem of biotic homogenization. Although the concept of biotic homogenization has been used to describe the process of replacement of local biota by exotic species (usually due to human activities, see [6]), biotic homogenization may also occur considering only native species [7,8]. For example, for a given set of sites (e.g., in a hydrographic basin), the decrease of beta diversity over time may be caused by local extinctions and increased occurrence of a few native species with certain traits (see Table 3 in [6]). Consistent with the traditional concept, anthropogenic environmental change may be the main cause of biotic homogenization. Nevertheless, according to Magurran et al. [5], “As all communities experience temporal turnover, one of the biggest challenges is distinguishing change that can be attributed to external factors, such as anthropogenic activities, from underlying natural change”.

In general, the list of prime factors and processes, not mutually exclusive, which could explain the variation in beta diversity includes environmental heterogeneity, spatial extent and productivity [9–11]. A particular set of locations may have higher beta diversity when compared with other sets of locations due to a higher environmental heterogeneity in the former. The different environmental conditions can alter the demographic rates of species differently, increasing the magnitudes of changes in species composition. In extreme cases of environmental differences (e.g., plankton communities in lakes with and without submerged plants), a small number of sites can be account for much of the total beta diversity [12]. Beta diversity may increase with spatial extent due to more heterogeneous environments and due to lower dispersal rates between sites separated by large geographical distances. Also, productive sites may have higher beta diversity because stochastic community assembly processes tend to be more important than deterministic ones [10].

Disentangling the relative role of these predictors on beta diversity variation is, nevertheless, difficult due to their interrelationship. For example, environmental heterogeneity is likely to be positively correlated with spatial extent, hindering the evaluation of the unique effects of environmental heterogeneity and dispersal on beta diversity. However, depending on the sampling design and the system investigated, some confounding factors can be ruled out. If, for example, community data are obtained at the same sites over time, then the effects of spatial extent on beta diversity can be ruled out because the geographic distances among sites would be maintained constant. In this context, hydroelectric reservoirs are excellent models for beta diversity studies due to high environmental heterogeneity along their longitudinal axes promoted by transport phenomena [13]. For example, different regions can be found at reservoirs: riverine, transitional and lacustrine. The riverine region is characterized by shorter water residence time. In general, in this region, turbidity is high and primary productivity is low owing to light limitation. The lacustrine region, on the other hand, tends to have the longest water residence time and primary productivity tends to be low due to nutrient limitation. Intermediate flow rates, water residence time, water transparency and nutrient availability, as well as higher primary productivity rates are, in general, expected at the transitional region [14]. This is, of course, an idealized description of the regions in a reservoir and the limits of these regions are dependent on hydrological variations. Most importantly, the level of environmental heterogeneity is expected to vary over time. During periods of higher environmental heterogeneity, one can envisage a stronger role of species sorting mechanisms and, therefore, an increased variation in community composition along the main axis of the reservoir (for a similar reasoning, see [15]).

There is a growing number of studies focusing on the relative roles of spatial and environmental processes on zooplankton community structuring (e.g. [16, 17, 18]). However, to the best of our knowledge, few studies have examined how the aforementioned factors relate to the variation in zooplankton community structure among a set sampling sites (e.g. [19, 20, 21]), which was defined by Anderson et al. [22] as a second type of beta diversity. It is important to emphasize that, to model this type of beta diversity, one needs first to sample multiple sites in different areas (e.g. watersheds) or multiple sites through time. With these data at hands, the second type of beta diversity can be estimated for each unit of analysis (e.g. watershed or time according to the examples cited above). This is the focus of our work. Specifically, we used a long-term dataset of zooplankton community in a tropical reservoir to test the hypothesis that beta diversity is positively related to environmental heterogeneity and productivity. Considering the high spatial heterogeneity in reservoirs [13, 14], we also predicted that beta diversity would be high over time. However, the levels of connectivity and environmental similarity between sites may vary depending on hydrological variations. Thus, we also expected a decrease in beta diversity during periods of high water level. This prediction is justified considering that increasing flow may cause the reduction of environmental heterogeneity, as well as increases in hydrological connectivity and passive dispersal rates.

## Material and methods

### Study area

This study was conducted in the Ribeirão das Lajes Reservoir (Rio de Janeiro State, Brazil). Ribeirão das Lajes Reservoir has a surface area (at the maximum water level) of 47.8 km^2^, a volume of 450 x 10^6^ m^3^ and an average depth of 15 m (maximum = 40 m). Water residence time is about 300 days and it has been considered an oligo-mesotrophic environment [23, 24]. Built in 1905, this reservoir is used for electricity generation and water supply. This research was part of the monitoring program commissioned by the electric energy concessionaire of the State of Rio de Janeiro (Light CORP) and did not involve endangered or protected species. Water level varies markedly (up to 8 m) and is generally correlated with precipitation. The lowest and the highest water levels are usually recorded in November (early rainy season) and April (end of rainy season), respectively. Thermal stratification occurs during most of the year at deeper regions and water column mixing (partial or complete) occurs only during winter months (June, July and August; [25]).

### Sampling

**Fig 1:**
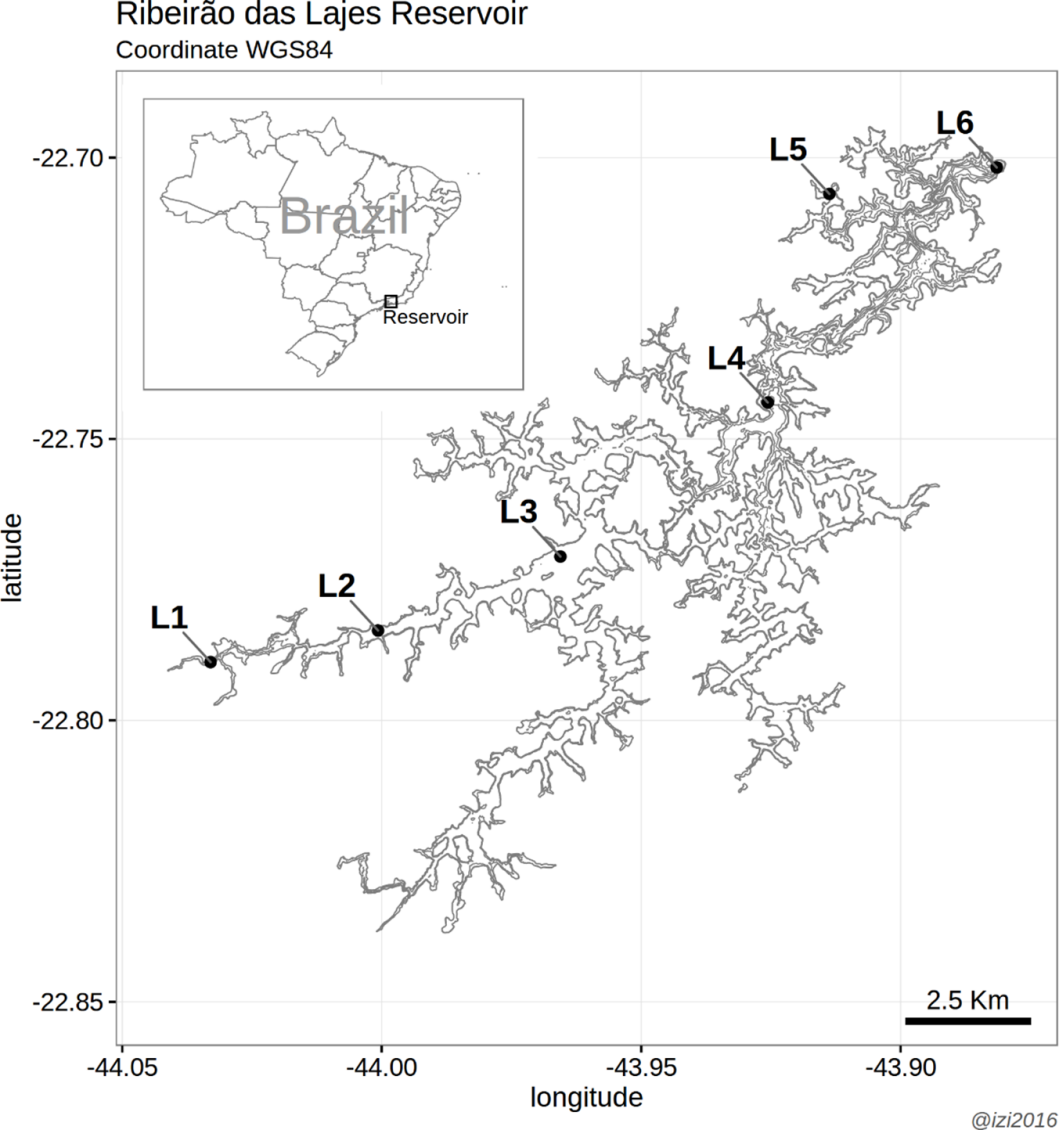
Study area (Ribeirão das Lajes Reservoir, municipality of Piraí, Rio de Janeiro State). The location of the sampling sites is also shown (L1: riverine region; L2, L3 and L4: transition region; L5: arm near the lacustrine region; L6: lacustrine region, near the dam).

Samples were taken monthly from November 2004 to December 2009, totaling 62 months at six sampling sites in the Ribeirão das Lajes Reservoir. To represent the environmental and biological variability of the reservoir, as much as possible, the sampling sites were distributed in the following way: the first sampling site (L1) was located at the riverine region of the reservoir; sites L2, L3, L4 and L6 (near the dam) were successively distributed along the longitudinal axis of the reservoir (from the riverine to the lacustrine regions); and site 5 (L5) was located at an arm near the lacustrine region (Fig 1). In general, these sampling sites differed mainly with respect to nutrient concentrations and water transparency (S1 Table).

Zooplankton samples were collected with a bucket (20 L) and by filtering twenty liters of subsurface water through a plankton net with 68 μm mesh size, according to the protocol established by the Rio de Janeiro State Environmental Institute (FEEMA / INEA). Given the selectivity of this mesh size, our results should be restricted to organisms larger than 68 μm [26]. Samples were preserved in formalin (4%) and buffered with sodium tetraborate (borax). Rotifera, Cladocera, and Testate amoebae were the zooplankton groups analyzed in this study. We used an optical microscope and standard slides for identification of the taxa. Identification followed Deflandre [27, 28], Koste [29], Vucetich [30], Nogrady et al. [31], Segers [32], Velho and Lansac-Tôha [33], Elmoor-Loureiro [34], Nogrady and Segers [35]. To estimate the density of each taxa (individuals/m^3^), we used a Sedgwick-Rafter counting cell, which was filled with sub-samples taken with a Hensen-Stempel pipette (1 mL). We consecutively analyzed the sub-samples until, at least, the enumeration of 150 individuals of the most abundant taxa. We analyzed the entire sub-samples to detect and enumerate rare species [36]. When necessary, for proper identification, the specimens were removed from the Sedgwick-Rafter cell, placed in standard glass slides and analyzed under microscope at 400x to 1000x magnification.

Dissolved oxygen, temperature, pH and conductivity were measured *in situ* using a multiparameter probe (YSI-85). Water transparency was estimated with a Secchi disk. Water samples were analyzed for orthophosphate, total phosphorous, nitrite, nitrate and ammonium according to APHA [37] and for chlorophyll-*a* according to Nusch and Palme [38].

### Beta diversity measures

Seven beta diversity measures were calculated assuming that different approaches can emphasize different data properties [22]. In general, the higher the value of an index in each month (considering the structure of our dataset), the higher the variation in community structure (when abundance data were used) or the higher the change in species composition among the six sites (for presence/absence data). Based on abundance data, a compositional dissimilarity matrix (between sites) was calculated using the Bray-Curtis coefficient [39] for each sampling month. The average of each Bray-Curtis dissimilarity matrix (βBC) was the first measure used to represent beta diversity in each month. The second measure of beta diversity (dBC) was the average Bray-Curtis dissimilarity from sampling sites to their group centroid (formed by the six sampling sites in a given month; [40]). Using presence-absence data, Sørensen (βSØR) and Simpson (βSIM) coefficients were calculated considering multiple sampling sites (see equations 5 and 6 in [41]). According to Baselga et al. [42], “A multiple-site index avoids (i) the loss of information concerning the number of species shared among three or more sites and (ii) the lack of independence between pairwise similarities due to the repetition of each site in several pairs” (see also [43]). βSIM, moreover, has the advantage of being independent of species richness and is the turnover component of βSØR measure [41–43]. The fifth measure was the nestedness component (βNES) of βSØR (βNES = βSØR- βSIM). The sixty measure (βRC) was calculated using the modified Raup-Crick index [44]. This index calculates the dissimilarity between samples using a null model approach. Chase et al. [44] explain the characteristics of the null model in detail, but the following sentence summarizes the main idea: “…, if *SS*_1,2_ is the observed number of shared species between localities 1 and 2, containing α_1_ and α_2_ species, respectively, βRC uses a randomization approach to estimate the probability of observing *SS*_1,2_ given repeated random draws of α_1_ and α_2_ species from a known species pool”. The main change of the Raup-Crick coefficient proposed by Chase et al. [44], which was used in this study, consisted of standardizing the coefficient to range from -1.0 to 1.0. A value close to zero indicates no difference from the null model, while a value approaching 1.0 suggests that the sites are more different from each other than expected by chance. Conversely, a value of -1.0 indicates that sites are more similar than expected by chance. Finally, the method described by Legendre & De Cáceres [12] was used to calculate the total beta diversity (total BD). These indexes were calculated in the R environment for statistical computing [45] using the functions *betadisper*, in the package *vegan* [46], and *beta*.*multi*, in the *betapart* package [43]. The modified index Raup-Crick was calculated using the R script provided in the supplementary material of Chase et al. [44]. The function *beta*.*div*, available in the supplementary material of Legendre and De Cáceres [12], was used to calculate the total beta diversity (for each month).

### Explanatory variables

Environmental heterogeneity (dC Env), for a given sampling month, was estimated according to the method proposed by Anderson et al. [40]. This method was applied to the standardized environmental dataset and using the average Euclidean distance matrix. The function *betadisper* was also used to estimate dC Env. The higher the values of dC Env, the higher the environmental differences among sampling sites in a given month. The mean chlorophyll-*a* concentration was used as a surrogate for productivity [9, 47]. Data on water level were obtained from the hydrological monitoring station of Light Energia CORP (S1 Table).

### Data analysis

**Fig 2:**
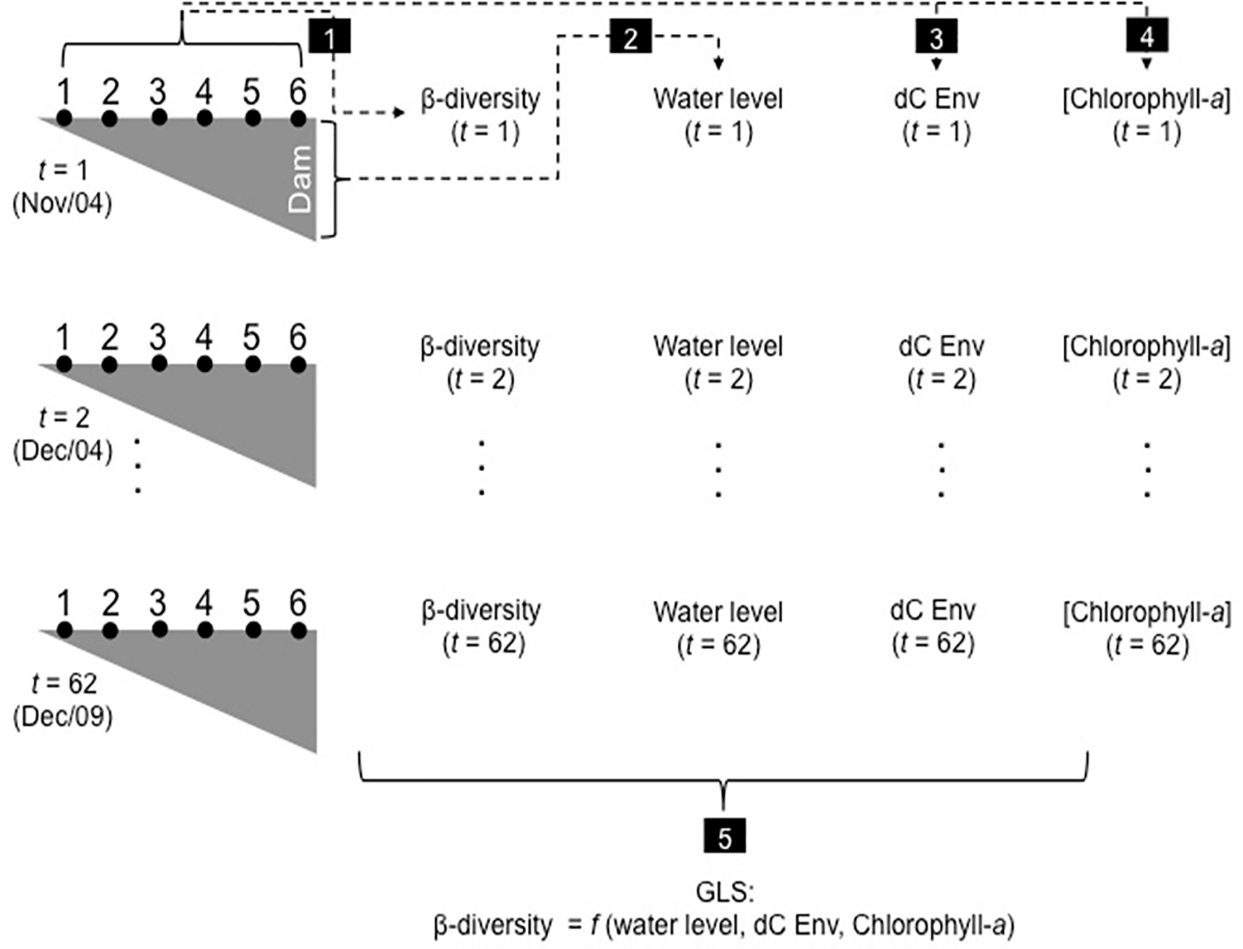
Schematic representation of our analytical framework. Environmental and zooplankton composition data, obtained monthly (from November/2004 to December/2009) at 6 sampling sites distributed along the major axis of the reservoir (•), were used to estimate, for each month, (1) beta diversity and (3) environmental heterogeneity (dC Env). Mean values of water level (2) and chlorophyll-a concentration (4), for each sampling month, were also recorded. The variable time was given as the chronological order of the sampling campaigns (5). A generalized least squares (GLS) model was used to model the temporal variation in beta diversity (6).

Generalized least squares models (GLS) were used to model the temporal variability in zooplankton beta diversity [48], while taking into account temporal autocorrelation in the data. Following Zuur et al. [48], we first fitted an ordinary least squares (OLS) model, without autocorrelation structure, in order to have a reference point. After, we specified three models allowing for residual autocorrelation: compound symmetry structure, autoregressive model of order 1 (ARMA(1, 0)) and autoregressive model of order 2 (ARMA(2, 0)). The compound symmetry structure assumes that residual correlation is the same independently of time lags. The autoregressive model of order 1 assumes that the residual at time *t* is a function of the residual at time *t* -1, whereas the autoregressive model of order 2 assumes that the residual at time *t* is a function of the residuals at time *t* -1 and *t*– 2 [48]. These models were compared using the Akaike Information Criterion (AIC). In all cases, the explanatory variables that represent our general hypothesis were included in the models (i.e., beta diversity is related to environmental heterogeneity (dC Env), chlorophyll-*a*, water level, and time). GLS models were estimated using the function *gls* of the nlme package [49]. Our analytical framework is summarized in Fig 2. All analyses were carried out using the entire biological dataset (i.e., including all taxa of rotifers, testate amoebae and cladocerans; S2 Table) and for each zooplankton group separately.

## Results

**Fig 3:**
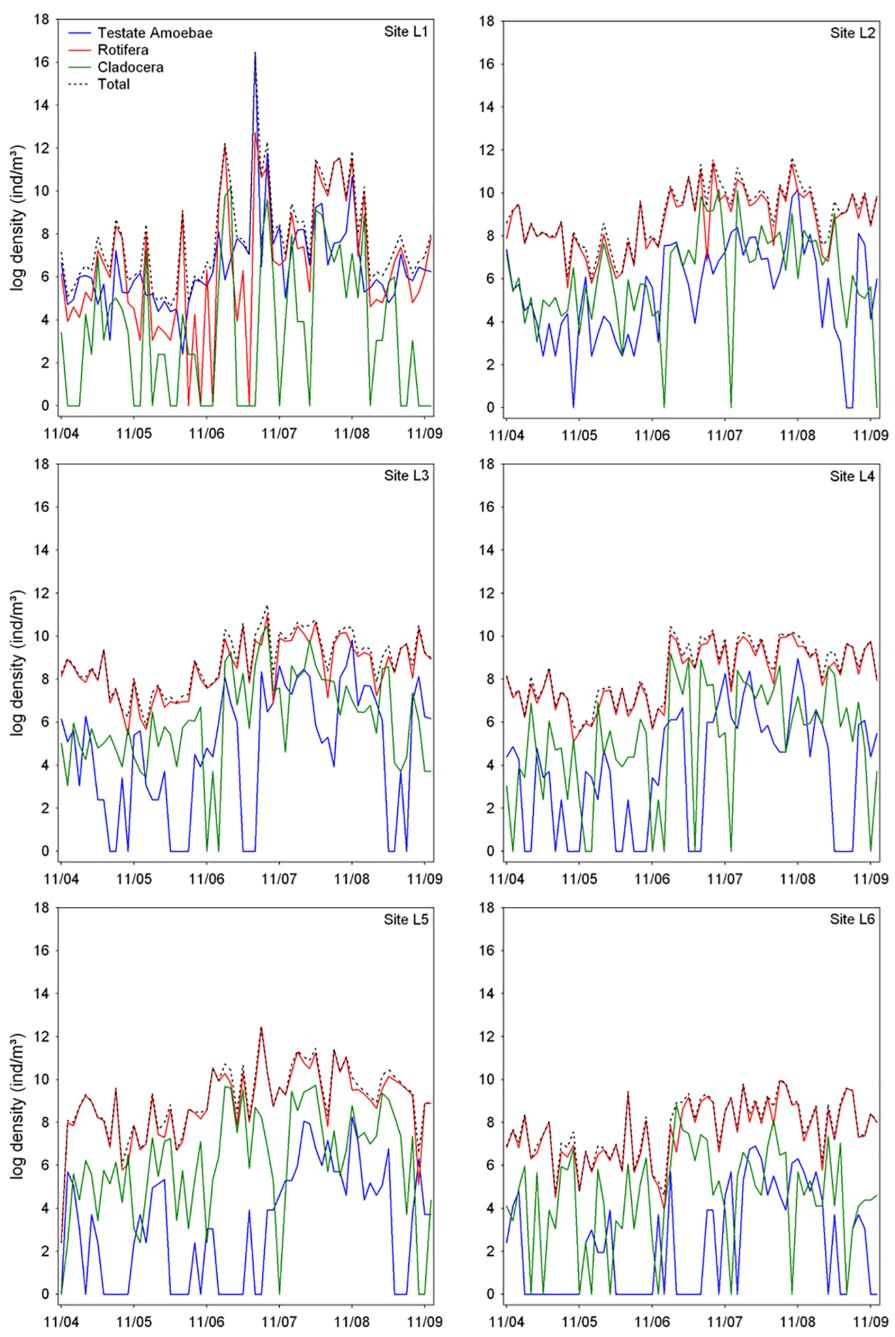
Temporal dynamics of zooplankton abundance. Shown are the time series for each zooplankton group and for the different sampling sites (Ribeirão das Lajes Reservoir; Brazil).

**Fig 4:**
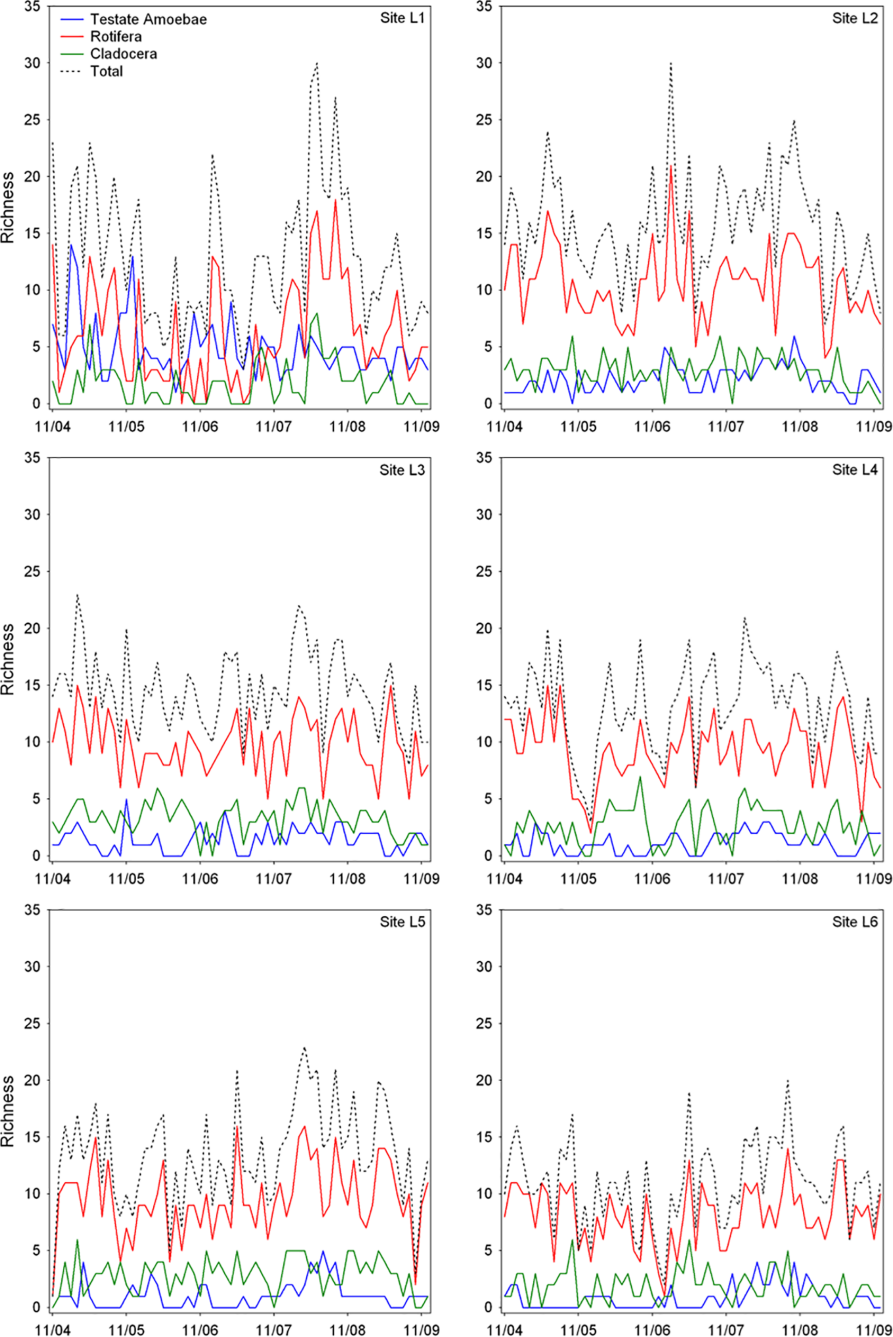
Temporal dynamics of zooplankton richness. Shown are the time series for each zooplankton group and for the different sampling sites (Ribeirão das Lajes Reservoir; Brazil).

We identified 161 taxa, 95 of rotifers, 37 of testate amoebae and 29 of cladocerans (S3 Table). The most common taxa during the study were *Difflugia* sp., *Conochilus unicornis*, *Ptygura* sp. and *Ceriodaphnia silvestrii*. Testate amoebae (particularly species of the genera *Centropyxis* and *Difflugia*) was the group with the highest abundance and number of taxa in the riverine region of the reservoir (site L1). At other sampling sites, there was an increase in the relative contribution of rotifers in terms of abundance and species richness. For example, *Ptygura* sp. was the most abundant taxa at site L2 and *Conochilus unicornis* at site L6. In general, sites L2, L3 and L5 showed the highest values of density and richness (Figs 3 and 4).

**Fig 5:**
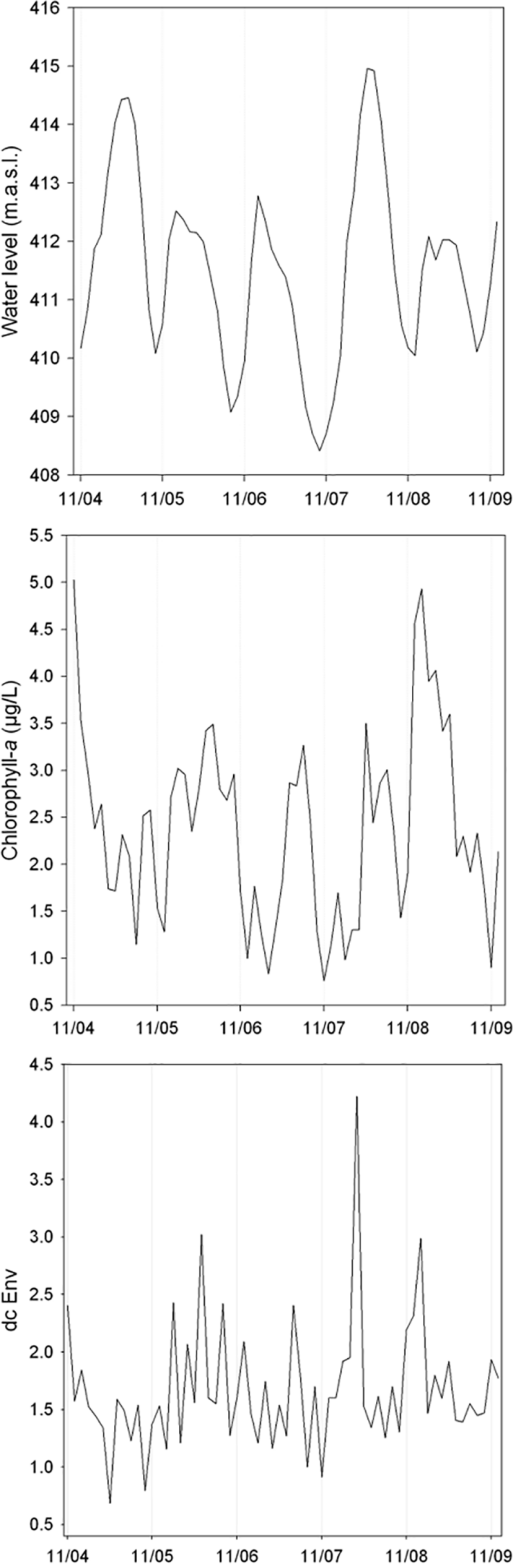
Temporal variation of the explanatory variables used in this study. (dC Env = environmental heterogeneity estimated by the method proposed by Anderson et al. [22]).

Water level varied seasonally during the study period (Fig 5), with the highest positive and negative temporal autocorrelation values for the first two lags and for the sixth lag, respectively (S4 Table). Chlorophyll-*a* showed high (positive) and significant temporal autocorrelation for the first two lags (Fig 5; S4 Table). On the other hand, no temporal autocorrelation was detected for environmental heterogeneity (dC Env; Fig 5 and S4 Table).

**Fig 6:**
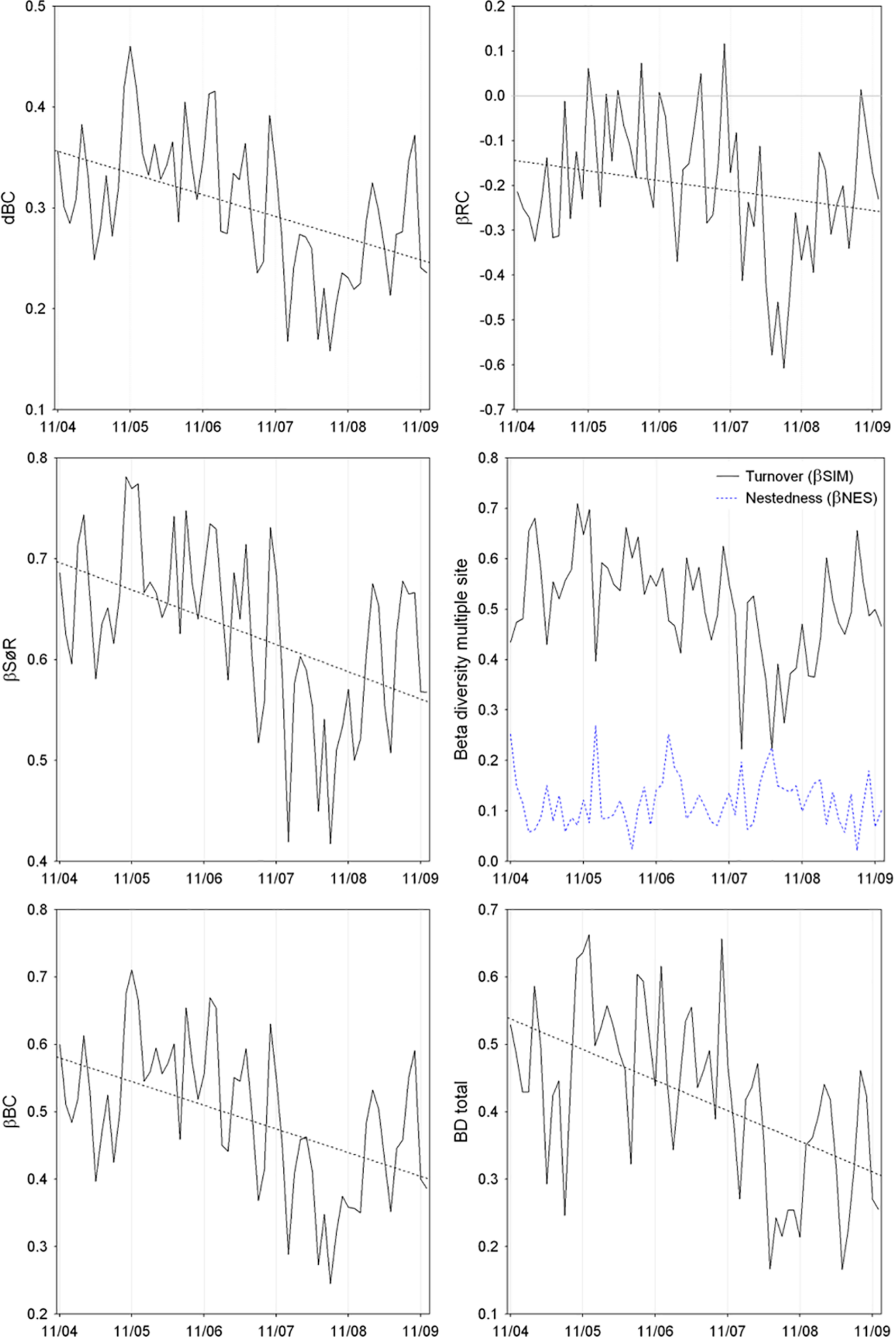
Temporal variation of zooplankton beta diversity at Ribeirão das Lajes Reservoir, Brazil. dBC: average distance to group centroid; βRC: modified Raup-Crick index; βSØR: Sørensen multiple site dissimilarity index; βSIM: Simpson multiple site dissimilarity index; βNES: Nestedness component, βBC: average of the Bray-Curtis dissimilarity matrix; BD total: total beta diversity. The horizontal line indicates the results expected by chance (for βRC).

Different beta diversity measures (average of the Bray-Curtis distance matrix, Sørensen and Simpson coefficients for multiple samples, average distance to group centroid, total BD and Raup-Crick coefficient) were strongly correlated to each other. Only βNES was weakly related with the beta diversity measures (Table 1, Fig 6). No seasonal patterns were identified and the time series indicated a downward trend between November 2004 and July 2008. After this last month, the beta diversity values increased and were similar to those measured at the beginning of time series (Fig 6). In addition to showing a similar temporal trend to that found for other beta diversity measures (Table 1), the Raup-Crick coefficient indicated that localities were more similar in terms of species composition than expected by chance in almost every month (i.e., low beta diversity; Fig 6). We also found that the turnover component (βSIM) was always higher than the nestedness component (βNES) of beta diversity (Fig 6).

**Table 1:** Pairwise Pearson’s correlation coefficients between beta diversity measures estimated for zooplankton communities in Ribeirão das Lajes Reservoir. βBC: average of the Bray-Curtis dissimilarity matrix; dBC: average distance to group the centroid group; βRC: modified Raup-Crick index; βSØR: Sørensen multiple site dissimilarity index; βSIM: Simpson multiple site dissimilarity index; βNES: Nestedness component; BD total: total beta diversity.

	βBC	βSør	βSim	βNes	dBC	βRC	BD total
βBC	1						
βSør	0.95	1					
βSim	0.78	0.87	1				
βNes	-0.05	-0.16	-0.62	1			
dBC	0.99	0.95	0.76	-0.02	1		
βRC	0.74	0.67	0.65	-0.24	0.73	1	
BD total	0.86	0.77	0.64	-0.08	0.85	0.66	1

Considering the strong correlations between beta diversity measures (Table 1) and the results obtained by Bennett et al. [35], only dBC and βNES were modeled as a function of the explanatory variables. The AICs indicate that the autoregressive models (ARMA(1,0) or ARMA(2,0)), for dBC, were substantially superior to the model without autocorrelation structure (OLS) or to the model assuming compound symmetry (Table 2). Models ARMA(1,0) and ARMA(2,0) were similarly supported as a basis for inference, given the data available (delta AIC = 0.10). According to these models, only the coefficient associated with time, indicating a decline in beta diversity over time, was statistically significant (Table 3). Residuals from these models were normally distributed (Shapiro-Wilk test; *W* = 0.99; *P* = 0.622 and *W* = 0.98, *P* = 0.598, for models ARMA(1,0) and ARMA(2,0), respectively). These results were not influenced by multicollinearity as indicated by the low Variance Inflation Factors (VIF _time_ = 1.01; VIF _water level_ = 1.01; VIF _Chlorophyll-a_ = 1.01; VIF _dC Env_ = 1.00). The best supported models (ARMA(1,0) and OLS) indicated that no explanatory variables were significantly associated with βNES (Tables 2 and 3).

**Table 2:** Akaike Information Criterion (AICc), delta AIC and Akaike weights for models with different autocorrelation structures (assuming no autocorrelation (OLS), ARMA(1,0), ARMA(2,0) and Compound Symmetry). **The Ordinary Least Squares (OLS) model does not allow for temporal autocorrelation.** The Akaike weights can be interpreted as the “relative likelihood of the model, given the data” [50]. dBC: average distance to the centroid group; βNES: Nestedness component.

Beta diversity measure	Models	AICc	delta AICc	Weight
dBC	ARMA(1,0)	-136.8	0.00	0.52
	ARMA(2,0)	-136.6	0.10	0.48
	OLS	-123.8	12.90	0.00
	Compound Symmetry	-121.3	15.50	0.00
βNes	ARMA(1,0)	-133.2	0.00	0.40
	OLS	-132.9	0.40	0.33
	ARMA(2,0)	-131.7	1.50	0.18
	Compound Symmetry	-130.3	2.90	0.09

**Table 3:** GLS models incorporating different autocorrelation structures (assuming no autocorrelation (OLS), ARMA(1,0) and (ARMA(2,0)) for zooplankton beta diversity (dBC and βNes) at Ribeirão das Lajes Reservoir, Brazil. *SE* = Standard Error; dC Env = Environmental heterogeneity; dBC: average distance to the centroid group; βNES: Nestedness component.

Beta diversity measure	Models	Variables	Coeff.	*SE*	*t*	*P*
dBC	ARMA(1,0)	(Intercept)	4.155	2.787	1.491	0.142
		Time	**-0.002**	**0.001**	**-2.685**	**0.010**
		Chlorophyll-a	-0.004	0.008	-0.428	0.670
		dC Env	0.004	0.011	0.360	0.720
		Water level	-0.009	0.007	-1.362	0.179
	ARMA(2,0)	(Intercept)	4.574	2.694	1.698	0.095
		Time	**-0.002**	**0.001**	**-3.356**	**0.001**
		Chlorophyll-a	0.001	0.008	0.107	0.915
		dC Env	0.003	0.010	0.304	0.762
		Water level	-0.010	0.007	-1.568	0.123
βNes	ARMA(1,0)	(Intercept)	-2.344	2.215	-1.058	0.295
		Time	0.000	0.000	0.144	0.886
		Chlorophyll-a	0.010	0.008	1.302	0.198
		dC Env	-0.004	0.012	-0.331	0.742
		Water level	0.006	0.005	1.104	0.274
	OLS	(Intercept)	-2.439	1.782	-1.368	0.177
		Time	0.000	0.000	0.342	0.734
		Chlorophyll-a	0.006	0.007	0.903	0.370
		dC Env	0.000	0.013	-0.039	0.969
		Water level	0.006	0.004	1.426	0.159

We also found that a temporal decline in beta diversity was the main pattern in our data when the analyses were repeated for each zooplankton group separately. However, the temporal variation in testate amoebae beta diversity was not significantly predicted by any of the explanatory variables we evaluated (S5 and S6 Tables).

## Discussion

In this study we evaluated the temporal variation in beta diversity (“variation in community structure among a set of sampling units”; [22]) of the zooplankton community at the Ribeirão das Lajes Reservoir over 62 months. Most of the previous studies that aimed to determine the relative importance of beta diversity correlates were carried out considering the spatial dimension (e.g., [9, 15, 51]). Specifically, in these studies, beta diversity was measured at different geographic areas bounded according to different criteria (e.g., ecoregion; see [51]) or for focal cells, in a map, considering adjacent cells [52,53]. For the first set of studies (i.e., assessing beta diversity in different regions), an important confounding factor is the relationship between spatial extent and environmental heterogeneity. This is a confounding factor because the larger the geographic distance between the farthest sampling sites in a region (i.e. spatial extent), the higher the environmental heterogeneity in that region. For the second set of studies, which mapped beta diversity, different caveats can be considered as, for instance, the arbitrariness of cell size and the problems with extent of occurrence data (Wallacean shortfall; [54]). Thus, ruling out the effect of spatial extent (but not necessarily the level of spatial connectivity—see below), as distances between sites were kept constant, is an advantage of our study design (see also [11]). Comparatively, our data could have had a great potential to unveil the main determinants of beta diversity.

Our results show, however, that some of the explanatory variables, commonly regarded as important in explaining beta diversity variation, were not significant predictors, at least for the environment studied here. Environmental heterogeneity, for example, has often been suggested as an important determinant of beta diversity (e.g., [9,55,56]). Thus, we expected that during periods of higher environmental heterogeneity we would find high beta diversity, as increases in environmental heterogeneity encompasses “an increase in the variety of environmental conditions to which different species are adapted, hence producing greater variation in species composition among localities within a region unit” [57]. However, such a relationship was not found, despite the high and temporally variable environmental heterogeneity (Fig 5). Thus, it is unlikely that our failure to detect a significant relationship between beta diversity and environmental heterogeneity was due to a lack of variability in our data. Although our measure of environmental heterogeneity included variables with known effects on zooplankton dynamics (e.g. [19, 58–60]), we cannot rule out, however, that we missed relevant variables. Another issue to be considered is the limitation in sampling organisms smaller than 68 μm, which may have influenced the characterization of the zooplankton community [26]. However, because we used the same methods during the entire monitoring program, we believe that our results are consistent for the zooplankton community larger than 68 μm.

Productivity had been thought to be an important determinant of beta diversity variation. According to Chase et al. [61], a positive relationship between beta diversity and productivity may arise because productivity itself is positively related to environmental heterogeneity, variance in species composition and the likelihood of communities to obtain multiple stable states. More recently, Chase [10] suggested that an increase in beta diversity with productivity occurs because, under this condition, stochastic assembly processes (e.g., ecological drift) are more important than deterministic processes (e.g. species sorting). This prediction assumes that different species can colonize more productive environments (low environmental filter caused by high productivity) and a significant role of priority effects (i.e. initial species composition influences the final composition). Despite experimental [10] and observational [11, 51, 61] evidences for a positive relationship between beta diversity and productivity, as well as the soundness of the arguments underlying it, our results did not support such a relationship. The low variability in chlorophyll-*a* concentration (monthly averages ranging from 0.76 μg/L to 5.03 μg/L) may explain our failure to detect a significant relationship between beta diversity and productivity in our study system. Also, negative relationships between beta diversity and productivity have been found considering wider gradients of chlorophyll-*a* [55]. An increase in productivity due to cultural eutrophication could lead to the dominance of a few eutrophic-tolerant species with the consequent decline in beta diversity [55]. Taken together, these results indicate that the direction of relationship between productivity and beta diversity (i.e., positive, negative or non-existent) may depend on several factors, including temporal/spatial scales, length of the gradient in productivity, biological group, type of study (observational versus experimental) and surrogate variable for productivity.

The role of hydrology on the dynamics of aquatic communities cannot be overstated [62–65]. In reservoirs, for example, the impacts of hydrological changes on biodiversity have been studied both downstream and upstream of dams [66–68]. We predicted a negative relationship between beta diversity and water level because during periods of high water level, the high influx of water into the reservoir would, simultaneously, increase the hydrological connectivity in the system and the rates of passive dispersal from the upstream to the downstream sites, as well as an increase in the environmental similarity between these sites. As a result of both mechanisms, a reduction in beta diversity would be expected. Environmental heterogeneity (dC Env) was not significantly correlated with water level (*r* = 0.033; *P* = 0.1280; cross-correlation analysis on differenced time series). Therefore, mass effects [69], instead of environmental homogenization, would more likely explain a significant relationship between water level and beta diversity. However, despite previous evidences in floodplain systems [70,71], our best-supported models did not show a significant relationship between beta diversity and water level. This result is unlikely to be explained by the low temporal variability in water level (Fig 5).

Our hypothesis that zooplankton beta diversity would be, in general, high because of the different regions along the major axis of the reservoir (i.e., riverine, intermediate and lacustrine), with different environmental characteristics, was not supported. Instead, the Raup-Crick metric indicated that zooplankton composition were recurrently more similar than expected by chance. According to Chase [44], this result would indicate a preponderant role of deterministic processes in the assembly of zooplankton communities in Ribeirão das Lajes Reservoir. Inferring processes from patterns is always questionable [72]. However, the low phytoplankton biomass allow us to infer that the low productivity (as proxied by chlorophyll-*a* concentration) is a mechanism (deterministic) of community assembly in this reservoir that cannot be discarded. This inference assumes that environmental filters (low productivity in our study) prevent that groups of species from the regional pool persist in the localities, resulting in more deterministic communities [73]. In addition, the hydrological connectivity between the sampling sites may have contributed to the low beta diversity in the reservoir [74,75].

We also detected a downward trend in beta diversity. First, this result indicates an ongoing dynamic in the reservoir, even after more than 100 years since its formation. It also weakens the concept of “reservoir stabilization”, which is often used by Brazilian scientists and technicians from the public and private sectors involved in biomonitoring programs. We agree that local processes causing temporal trends in reservoirs are likely to be reduced over time (e.g. decomposition of flooded vegetal biomass and increased abundance of species favored by a lacustrine environment). However, it is unlikely that other processes interfering in the dynamics of reservoirs stabilize over time (e.g., land use in the watershed). Second, several scenarios, involving a balance between extinction and invasion rates, can be envisioned to explain the biotic homogenization process that we detected. Although we cannot identify the most likely scenario (among those listed by Olden and Poff [7]), our results are consistent with those obtained in many aquatic ecosystems worldwide by also demonstrating a decline in beta diversity over time (e.g., [76,77]).

In conclusion, we detected a process of biotic homogenization (i.e., beta diversity decline), which is a cause of concern worldwide. We also suggest that the low zooplankton beta diversity over time may be accounted for by the low trophic status of the reservoir. However, our results revealed that predicting beta diversity was challenging and that we are far from a reliable list of beta diversity predictors (see also [78]). Given the importance of beta diversity to inform biodiversity conservation [79] and biomonitoring [80, 81], we thus suggest the need to reevaluate predictions or, at least, to search for better surrogates of the processes that hypothetically control beta diversity variation. In addition, we suggest the need of further studies to better understand the main determinants of variation in beta diversity over time. We believe that these studies are already feasible considering mainly the data accrued in long-term ecological studies around the world.

## Supporting information

S1 Table Environmental characterization of each sampling site in the Ribeirão das Lajes Reservoir (Rio de Janeiro State, Brazil). Data were obtained from November/2004 to December/2009.
(DOCX)

S2 Table Dataset used in this study. (Ch-a = Chlorophyll-*a*; dC Env = Environmental heterogeneity; dBC = Average distance to group centroid (Bray-Curtis); teca = Testate Amoebae; roti = Rotifera; clado = Cladocera)
(DOCX)

S3 Table List of zooplankton taxa found in each sampling site in the Ribeirão das Lajes Reservoir (Rio de Janeiro State, Brazil). (DOCX)

S4 Table Temporal autocorrelation analysis of the explanatory variables used in this study. dC Env = Environmental heterogeneity.
(DOCX)

S5 Table Akaike Information Criterion (AIC_c_), delta AIC and Akaike weights for models with different autocorrelation structures (assuming no autocorrelation (OLS), ARMA(1,0), ARMA(2,0) and Compound Symmetry). dBC = average distance to group centroid; βNES = Nestedness component. Models were run for each zooplankton group separately.
(DOCX)

S6 Table Best selected models for each zooplankton group beta diversity (dBC and βNes) at Ribeirão das Lajes Reservoir, Brazil (see Table E). *SE* = Standard Error; dC Env = Environmental heterogeneity; dBC = average distance to group centroid; βNES = Nestedness component.
(DOCX)
