# Supplementary material for: Predicting temporal variation in zooplankton beta diversity is challenging
Source: PLoS One. 2017 Nov 2;12(11):e0187499. doi: 10.1371/journal.pone.0187499 (PMC5667886; doi:10.1371/journal.pone.0187499)
Supplement: S1 Table — Data were obtained from November/2004 to December/2009. (DOCX) [file pone.0187499.s001.docx]

**S1 Table.** **Environmental characterization of each sampling site in the Ribeirão das Lajes Reservoir (Rio de Janeiro State, Brazil).** Data were obtained from November/2004 to December/2009.

|  |  | **L1** | **L2** | **L3** | **L4** | **L5** | **L6** |
| --- | --- | --- | --- | --- | --- | --- | --- |
| Temperature (ºC) | min | 16.6 | 21.0 | 21.1 | 21.3 | 21.2 | 20.3 |
|  | med | 22.3 | 26.1 | 26.1 | 26.3 | 26.1 | 25.6 |
|  | max | 32.6 | 30.6 | 31.5 | 30.8 | 31.0 | 30.4 |
| pH | min | 5.1 | 5.3 | 5.8 | 5.6 | 5.5 | 5.6 |
|  | med | 6.3 | 6.9 | 7.0 | 6.8 | 6.7 | 6.6 |
|  | max | 7.1 | 8.6 | 8.6 | 8.7 | 8.6 | 8.0 |
| D.O. (mg.L^-1^ O_2_) | min | 4.8 | 4.4 | 4.3 | 4.4 | 4.1 | 3.8 |
|  | med | 8.4 | 8.1 | 7.9 | 7.7 | 7.3 | 7.6 |
|  | max | 12.7 | 12.8 | 12.6 | 12.2 | 12.3 | 11.7 |
| Conductivity (μS.cm^-1^) | min | 14.0 | 24.7 | 21.9 | 20.8 | 20.0 | 18.8 |
|  | med | 24.5 | 28.5 | 28.3 | 28.0 | 29.3 | 27.6 |
|  | max | 34.3 | 34.0 | 33.0 | 32.0 | 33.3 | 32.0 |
| Transparency (m) | min | 0.2 | 0.9 | 1.2 | 1.2 | 1.3 | 2.3 |
|  | med | 1.6 | 2.8 | 3.4 | 3.7 | 2.8 | 3.8 |
|  | max | 3.8 | 4.5 | 5.5 | 5.5 | 5.0 | 6.4 |
| Nitrite (μg.L^-1^ N) | min | ND | ND | ND | ND | ND | ND |
|  | med | 14.9 | 8.6 | 7.4 | 6.5 | 9.7 | 6.9 |
|  | max | 105.4 | 30.0 | 32.5 | 21.7 | 32.5 | 25.8 |
| Nitrate (μg.L^-1^ N) | min | 7.1 | 5.5 | 6.3 | 6.0 | 6.2 | 6.1 |
|  | med | 219.4 | 120.1 | 103.2 | 109.0 | 122.1 | 108.0 |
|  | max | 723.1 | 544.1 | 469.0 | 524.0 | 556.0 | 646.3 |
| Ammonium (μg.L^-1^ N) | min | 7.7 | 8.8 | 5.0 | 3.2 | 6.7 | 10.4 |
|  | med | 298.1 | 192.4 | 143.5 | 142.0 | 243.8 | 233.6 |
|  | max | 1787.5 | 743.5 | 632.1 | 436.6 | 883.0 | 1434.8 |
| Orthophosphate (μg.L^-1^ P) | min | 2.1 | 1.3 | 1.0 | 1.4 | 1.0 | 1.3 |
|  | med | 10.6 | 11.5 | 7.9 | 7.6 | 10.2 | 8.4 |
|  | max | 34.0 | 77.9 | 18.0 | 21.7 | 50.2 | 18.2 |
| total-P (μg.L^-1^ P) | min | 8.5 | 5.9 | 2.5 | 1.2 | 8.4 | 1.5 |
|  | med | 43.9 | 26.7 | 16.9 | 14.7 | 27.3 | 17.4 |
|  | max | 158.7 | 198.6 | 44.7 | 62.6 | 52.6 | 51.5 |
| Chlorophyll-*a*  (μg.L^-1^) | min | 0.3 | 0.2 | 0.3 | 0.1 | 0.4 | 0.1 |
|  | med | 2.3 | 2.5 | 2.2 | 1.5 | 4.0 | 1.4 |
|  | max | 8.2 | 5.9 | 7.4 | 5.6 | 9.4 | 5.6 |
| Depth | med | 5.0 | 15.0 | 20.0 | 30.0 | 15.0 | 35.0 |
