# Supplementary material for: Predicting temporal variation in zooplankton beta diversity is challenging
Source: PLoS One. 2017 Nov 2;12(11):e0187499. doi: 10.1371/journal.pone.0187499 (PMC5667886; doi:10.1371/journal.pone.0187499)
Supplement: S2 Table — (Ch-a = Chlorophyll-a; dC Env = Environmental heterogeneity; dBC = Average distance to group centroid (Bray-Curtis); teca = Testate Amoebae; roti = Rotifera; clado = Cladocera) (DOCX) [file pone.0187499.s002.docx]

**S2 Table. Dataset used in this study.** (Ch-a= Chlorophyll-*a*; dC Env = Environmental heterogeneity; dBC= Average distance to group centroid (Bray-Curtis); teca= Testate Amoebae; roti= Rotifera; clado= Cladocera)

|  | **water level**  **(m.a.s.l.)** | **Ch-a**  **(μg/L)** | **dC Env** | **Time** | **dBC teca** | **dBC roti** | **dBC clado** | **dBC teca+roti+clado** |
| --- | --- | --- | --- | --- | --- | --- | --- | --- |
| **Nov-04** | 410.2 | 5.03 | 2.41 | 1 | 0.393 | 0.330 | 0.381 | 0.357 |
| **Dec-04** | 410.8 | 3.54 | 1.58 | 2 | 0.236 | 0.294 | 0.406 | 0.301 |
| **Jan-05** | 411.9 | 3 | 1.85 | 3 | 0.238 | 0.290 | 0.180 | 0.285 |
| **Feb-05** | 412.1 | 2.38 | 1.53 | 4 | 0.345 | 0.254 | 0.429 | 0.308 |
| **Mar-05** | 413.2 | 2.64 | 1.44 | 5 | 0.436 | 0.348 | 0.348 | 0.383 |
| **Apr-05** | 414 | 1.74 | 1.34 | 6 | 0.464 | 0.286 | 0.448 | 0.330 |
| **May-05** | 414.4 | 1.72 | 0.68 | 7 | 0.459 | 0.197 | 0.352 | 0.249 |
| **Jun-05** | 414.5 | 2.32 | 1.59 | 8 | 0.433 | 0.253 | 0.340 | 0.279 |
| **Jul-05** | 414 | 2.08 | 1.49 | 9 | 0.500 | 0.372 | 0.162 | 0.332 |
| **Aug-05** | 412.6 | 1.15 | 1.23 | 10 | 0.577 | 0.253 | 0.254 | 0.272 |
| **Sep-05** | 410.8 | 2.52 | 1.54 | 11 | 0.507 | 0.310 | 0.253 | 0.319 |
| **Oct-05** | 410.1 | 2.58 | 0.80 | 12 | 0.000 | 0.480 | 0.330 | 0.421 |
| **Nov-05** | 410.6 | 1.53 | 1.37 | 13 | 0.493 | 0.436 | 0.398 | 0.460 |
| **Dec-05** | 412 | 1.28 | 1.53 | 14 | 0.412 | 0.402 | 0.416 | 0.417 |
| **Jan-06** | 412.5 | 2.72 | 1.16 | 15 | 0.166 | 0.353 | 0.317 | 0.354 |
| **Feb-06** | 412.4 | 3.02 | 2.43 | 16 | 0.366 | 0.337 | 0.224 | 0.332 |
| **Mar-06** | 412.2 | 2.96 | 1.21 | 17 | 0.370 | 0.336 | 0.409 | 0.363 |
| **Apr-06** | 412.2 | 2.35 | 2.07 | 18 | 0.403 | 0.280 | 0.348 | 0.329 |
| **May-06** | 412 | 2.8 | 1.56 | 19 | 0.166 | 0.322 | 0.275 | 0.342 |
| **Jun-06** | 411.4 | 3.42 | 3.02 | 20 | 0.500 | 0.343 | 0.297 | 0.366 |
| **Jul-06** | 410.8 | 3.49 | 1.60 | 21 | 0.333 | 0.261 | 0.320 | 0.286 |
| **Aug-06** | 409.8 | 2.8 | 1.55 | 22 | 0.319 | 0.357 | 0.538 | 0.405 |
| **Sep-06** | 409.1 | 2.68 | 2.42 | 23 | 0.449 | 0.338 | 0.349 | 0.349 |
| **Oct-06** | 409.3 | 2.96 | 1.28 | 24 | 0.500 | 0.149 | 0.201 | 0.308 |
| **Nov-06** | 409.9 | 1.7 | 1.61 | 25 | 0.444 | 0.303 | 0.479 | 0.347 |
| **Dec-06** | 411.7 | 1 | 2.09 | 26 | 0.468 | 0.273 | 0.363 | 0.413 |
| **Jan-07** | 412.8 | 1.77 | 1.46 | 27 | 0.355 | 0.376 | 0.304 | 0.416 |
| **Feb-07** | 412.4 | 1.25 | 1.21 | 28 | 0.354 | 0.249 | 0.286 | 0.277 |
| **Mar-07** | 411.9 | 0.83 | 1.75 | 29 | 0.325 | 0.242 | 0.239 | 0.275 |
| **Apr-07** | 411.6 | 1.32 | 1.17 | 30 | 0.341 | 0.333 | 0.174 | 0.334 |
| **May-07** | 411.4 | 1.83 | 1.54 | 31 | 0.302 | 0.315 | 0.239 | 0.328 |
| **Jun-07** | 410.9 | 2.87 | 1.27 | 32 | 0.494 | 0.223 | 0.218 | 0.364 |
| **Jul-07** | 410 | 2.83 | 2.41 | 33 | 0.435 | 0.290 | 0.119 | 0.290 |
| **Aug-07** | 409.2 | 3.27 | 1.78 | 34 | 0.334 | 0.229 | 0.198 | 0.236 |
| **Sep-07** | 408.7 | 2.43 | 1.00 | 35 | 0.488 | 0.218 | 0.278 | 0.246 |
| **Oct-07** | 408.4 | 1.28 | 1.70 | 36 | 0.270 | 0.392 | 0.479 | 0.392 |
| **Nov-07** | 408.7 | 0.76 | 0.91 | 37 | 0.402 | 0.302 | 0.413 | 0.341 |
| **Dec-07** | 409.2 | 1.15 | 1.60 | 38 | 0.293 | 0.254 | 0.404 | 0.273 |
| **Jan-08** | 410 | 1.7 | 1.60 | 39 | 0.285 | 0.144 | 0.160 | 0.168 |
| **Feb-08** | 412 | 0.99 | 1.92 | 40 | 0.273 | 0.219 | 0.273 | 0.241 |
| **Mar-08** | 412.8 | 1.3 | 1.95 | 41 | 0.342 | 0.253 | 0.313 | 0.274 |
| **Apr-08** | 414.2 | 1.3 | 4.22 | 42 | 0.199 | 0.255 | 0.262 | 0.271 |
| **May-08** | 415 | 3.5 | 1.52 | 43 | 0.262 | 0.241 | 0.313 | 0.260 |
| **Jun-08** | 414.9 | 2.44 | 1.34 | 44 | 0.218 | 0.150 | 0.212 | 0.170 |
| **Jul-08** | 414.1 | 2.87 | 1.62 | 45 | 0.195 | 0.199 | 0.266 | 0.220 |
| **Aug-08** | 412.8 | 3.01 | 1.25 | 46 | 0.287 | 0.141 | 0.170 | 0.158 |
| **Sep-08** | 411.5 | 2.37 | 1.70 | 47 | 0.293 | 0.182 | 0.266 | 0.204 |
| **Oct-08** | 410.6 | 1.43 | 1.31 | 48 | 0.364 | 0.195 | 0.257 | 0.236 |
| **Nov-08** | 410.2 | 1.9 | 2.19 | 49 | 0.240 | 0.201 | 0.309 | 0.231 |
| **Dec-08** | 410 | 4.56 | 2.31 | 50 | 0.252 | 0.209 | 0.254 | 0.220 |
| **Jan-09** | 411.5 | 4.93 | 2.99 | 51 | 0.239 | 0.226 | 0.210 | 0.225 |
| **Feb-09** | 412.1 | 3.95 | 1.47 | 52 | 0.270 | 0.268 | 0.257 | 0.288 |
| **Mar-09** | 411.7 | 4.07 | 1.80 | 53 | 0.330 | 0.333 | 0.323 | 0.325 |
| **Apr-09** | 412 | 3.42 | 1.60 | 54 | 0.238 | 0.314 | 0.331 | 0.298 |
| **May-09** | 412 | 3.6 | 1.92 | 55 | 0.520 | 0.240 | 0.283 | 0.261 |
| **Jun-09** | 411.9 | 2.08 | 1.41 | 56 | 0.240 | 0.204 | 0.232 | 0.214 |
| **Jul-09** | 411.4 | 2.3 | 1.39 | 57 | 0.355 | 0.225 | 0.350 | 0.274 |
| **Aug-09** | 410.8 | 1.92 | 1.55 | 58 | 0.500 | 0.239 | 0.459 | 0.277 |
| **Sep-09** | 410.1 | 2.33 | 1.45 | 59 | 0.465 | 0.342 | 0.335 | 0.347 |
| **Oct-09** | 410.4 | 1.75 | 1.47 | 60 | 0.406 | 0.358 | 0.157 | 0.372 |
| **Nov-09** | 411.3 | 0.9 | 1.94 | 61 | 0.410 | 0.199 | 0.091 | 0.241 |
| **Dec-09** | 412.3 | 2.13 | 1.77 | 62 | 0.486 | 0.201 | 0.048 | 0.236 |
