# Supplementary material for: Predicting temporal variation in zooplankton beta diversity is challenging
Source: PLoS One. 2017 Nov 2;12(11):e0187499. doi: 10.1371/journal.pone.0187499 (PMC5667886; doi:10.1371/journal.pone.0187499)
Supplement: S3 Table — (DOCX) [file pone.0187499.s003.docx]

**S3 Table. List of zooplankton taxa found in each sampling site in the Ribeirão das Lajes Reservoir (Rio de Janeiro State, Brazil).**

| **Zooplankton taxa** | **L1** | **L2** | **L3** | **L4** | **L5** | **L6** |
| --- | --- | --- | --- | --- | --- | --- |
| **Testate Amoebae** |  |  |  |  |  |  |
| *Arcella conica* (Playfair, 1918) | **X** |  |  | **X** |  |  |
| *Arcella* cf. *costata* Ehrenberg, 1847 | **X** | **X** | **X** | **X** | **X** | **X** |
| *Arcella dentata* Ehrenberg, 1830 | **X** | **X** | **X** |  | **X** |  |
| *Arcella* cf. *discoides* Ehrenberg, 1843 | **X** | **X** | **X** |  |  |  |
| *Arcella* cf. *gibbosa* Penard, 1890 | **X** |  | **X** | **X** | **X** | **X** |
| *Arcella hemisphaerica* Perty, 1852 | **X** |  |  |  |  |  |
| *Arcella vulgaris* Ehrenberg, 1830 | **X** | **X** | **X** | **X** | **X** | **X** |
| *Arcella* sp. | **X** | **X** | **X** | **X** |  | **X** |
| *Centropyxis aculeata* (Ehrenberg, 1838) | **X** | **X** | **X** | **X** | **X** | **X** |
| *Centropyxis aculeata* var. *oblonga* Deflandre, 1929 | **X** |  |  |  |  |  |
| *Centropyxis* cf. *aerophila* Deflandre, 1929 | **X** |  |  |  |  |  |
| *Centropyxis* cf. *constricta* (Ehrenberg, 1841) | **X** |  |  |  |  |  |
| *Centropyxis* cf. *ecornis* (Ehrenberg, 1841) | **X** |  |  |  |  |  |
| *Centropyxis gibba* Deflandre, 1929 | **X** |  |  |  |  |  |
| *Centropyxis* cf. *hirsuta* Deflandre, 1929 | **X** |  |  |  |  |  |
| *Centropyxis* cf. *marsupiformis* (Wallich, 1864) | **X** |  |  |  |  |  |
| *Centropyxis* cf. *minuta* Deflandre, 1929 | **X** |  |  |  |  |  |
| *Centropyxis platystoma* (Pénard, 1890) | **X** |  |  |  |  |  |
| *Centropyxis* sp. | **X** |  |  |  |  |  |
| Cyclopyxis stellata (Wailes, 1927) |  | **X** |  |  |  |  |
| *Cucurbitella* sp. | **X** |  |  |  |  |  |
| *Difflugia angulostoma* Gauthier-Lièvre & Thomas, 1958 | **X** |  |  |  |  |  |
| *Difflugia corona* Wallich, 1864 | **X** | **X** | **X** | **X** | **X** |  |
| *Difflugia gramen* Pénard, 1902 | **X** | **X** | **X** | **X** | **X** | **X** |
| *Difflugia* cf. *lobostoma* Leidy, 1879 | **X** | **X** | **X** |  |  |  |
| *Difflugia lobostoma* var. *multilobata* Gauthier-Lièvre & Thomas, 1958 |  | **X** |  | **X** |  |  |
| *Difflugia oblonga* Ehrenberg, 1838 | **X** |  | **X** |  |  |  |
| *Difflugia pseudogramen* Gauthier-Lièvre & Thomas 1960 | **X** |  |  |  |  |  |
| *Difflugia turbeculata* (Wallich, 1864) | **X** | **X** | **X** | **X** |  |  |
| *Difflugia urceolata* Carter, 1864 |  |  |  |  |  |  |
| *Difflugia* sp. | **X** | **X** | **X** | **X** | **X** | **X** |
| *Euglypha* cf. *turbeculata* Dujardin, 1841 | **X** |  |  |  |  |  |
| *Euglypha* sp. | **X** |  |  |  |  |  |
| *Lesquereusia modesta* Rhumbler, 1896 | **X** |  |  |  |  |  |
| *Lesqueresia spiralis* (Ehrenberg, 1840) | **X** |  |  |  |  |  |
| *Lesquereusia* sp. | **X** | **X** |  |  | **X** | **X** |
| *Protocucurbitella* sp. | **X** |  | **X** |  |  |  |

| **Zooplankton taxa (cont.)** | **L1** | **L2** | **L3** | **L4** | **L5** | **L6** |
| --- | --- | --- | --- | --- | --- | --- |
| **Rotifera** |  |  |  |  |  |  |
| *Ascomorpha agilis* Zacharias, 1893 |  | **X** |  | **X** |  | **X** |
| *Ascomorpha ecaudis* Perty, 1850 | **X** | **X** | **X** | **X** | **X** | **X** |
| *Ascomorpha saltans* Bartsch, 1870 | **X** | **X** | **X** | **X** | **X** | **X** |
| *Ascomorpha* sp. | **X** | **X** | **X** | **X** | **X** | **X** |
| *Asplanchna* sp. | **X** | **X** | **X** | **X** | **X** | **X** |
| *Brachionus angularis* Gosse, 1851 | **X** | **X** | **X** | **X** |  |  |
| *Brachionus bidentatus* Anderson, 1889 |  |  |  |  | **X** |  |
| *Brachionus calicyflorus* Pallas, 1766 |  | **X** |  |  |  |  |
| *Brachionus caudatus* Barrois & Daday, 1885 |  | **X** |  | **X** |  |  |
| *Brachionus dolabratus* Harring, 1914 |  | **X** |  |  | **X** |  |
| *Brachionus falcatus* Zacharias, 1898 | **X** | **X** | **X** | **X** | **X** | **X** |
| *Brachionus gilardi* Hauer, 1966 |  |  |  | **X** |  |  |
| *Brachionus* cf. *havanaensis* Rousselet, 1911 |  |  |  |  |  | **X** |
| *Brachionus plicatilis* (Müller, 1786) |  |  |  | **X** |  |  |
| *Brachionus quadridentatus* Hermann, 1783 |  |  |  |  | **X** |  |
| *Brachionus* sp. |  | **X** |  |  |  | **X** |
| *Cephalodella* sp. | **X** | **X** |  |  | **X** |  |
| *Collotheca mutabilis* (Hudson, 1885) | **X** | **X** | **X** | **X** | **X** | **X** |
| *Collotheca ornata* (Ehrenberg, 1832) | **X** | **X** | **X** | **X** | **X** | **X** |
| *Collotheca* sp. | **X** | **X** | **X** | **X** | **X** | **X** |
| *Conochilus coenobasis* (Skorikov, 1914) | **X** | **X** | **X** | **X** | **X** | **X** |
| *Conochilus natans* (Seligo, 1900) |  | **X** |  | **X** | **X** | **X** |
| *Conochilus unicornis* Rousselet, 1892 | **X** | **X** | **X** | **X** | **X** | **X** |
| *Conochilus* sp. | **X** |  |  |  |  |  |
| *Dipleuchlanis* sp. | **X** |  | **X** |  |  |  |
| *Epiphanes* sp. |  |  |  | **X** |  |  |
| *Euchlanis dilatata* Ehrenberg, 1832 | **X** | **X** |  | **X** |  |  |
| *Euchlanis* cf *meneta* Myers, 1930 |  |  | **X** |  |  |  |
| *Euchlanis oropha* Gosse, 1887 | **X** | **X** |  | **X** |  |  |
| *Euchlanis* spp. | **X** | **X** | **X** | **X** | **X** | **X** |
| *Filinia longiseta* (Ehrenberg, 1834) | **X** | **X** |  |  |  |  |
| *Filinia opoliensis* (Zacharias, 1898) | **X** | **X** | **X** | **X** | **X** | **X** |
| *Filinia pejleri* Hutchinson, 1964 |  | **X** |  |  |  |  |
| *Filinia* sp. | **X** | **X** | **X** | **X** | **X** |  |
| *Gastropus* sp. | **X** | **X** | **X** | **X** |  |  |
| *Hexarthra* sp. | **X** | **X** | **X** | **X** | **X** | **X** |
| *Kellicottia bostoniensis* (Rousselet, 1908) | **X** | **X** | **X** | **X** | **X** | **X** |
| *Kellicottia longispina* (Kellicott, 1879) |  |  |  | **X** |  |  |
| *Kellicottia* sp. |  | **X** |  |  |  |  |
| *Keratella americana* Carlin, 1943 | **X** | **X** | **X** | **X** | **X** | **X** |
| *Keratella cochlearis* (Gosse, 1851) | **X** | **X** | **X** | **X** | **X** | **X** |
| *Keratella tecta* (Gosse, 1851) |  | **X** |  |  |  |  |
| *Keratella tropica* (Apstein, 1907) | **X** | **X** |  |  |  |  |
| *Lacinularia* sp. |  | **X** |  |  |  |  |
| *Lecane bulla* (Gosse, 1851) | **X** | **X** | **X** | **X** | **X** | **X** |
| *Lecane cornuta* (Müller, 1786) | **X** | **X** |  |  | **X** | **X** |

| **Zooplankton taxa (cont.)** | **L1** | **L2** | **L3** | **L4** | **L5** | **L6** |
| --- | --- | --- | --- | --- | --- | --- |
| **Rotifera (cont.)** |  |  |  |  |  |  |
| *Lecane curvicornis* (Murray, 1913) | **X** | **X** |  | **X** | **X** | **X** |
| *Lecane elsa* Hauer, 1931 |  | **X** |  |  |  |  |
| *Lecane hornemanni* (Ehrenberg, 1834) |  |  |  |  | **X** |  |
| *Lecane leontina* (Turner, 1892) | **X** |  |  |  | **X** |  |
| *Lecane ludwigii* (Eckstein, 1883) | **X** | **X** |  |  |  |  |
| *Lecane luna* (Müller, 1776) | **X** | **X** | **X** |  | **X** | **X** |
| *Lecane lunaris* (Ehrenberg, 1832) | **X** | **X** | **X** | **X** | **X** | **X** |
| *Lecane lunaris crenata* (Harring, 1913) |  |  | **X** |  |  |  |
| *Lecane melini* Thomasson, 1953 |  | **X** |  |  |  |  |
| *Lecane papuana* (Murray, 1913) | **X** | **X** |  |  |  |  |
| *Lecane signifera* (Jennings, 1896) | **X** | **X** | **X** | **X** | **X** |  |
| *Lecane stenroosi* (Meissner, 1908) | **X** |  |  |  |  |  |
| *Lecane thienemanni* (Hauer, 1938) | **X** |  |  |  |  |  |
| *Lecane* sp1. | **X** | **X** | **X** |  |  |  |
| *Lecane* sp2. |  | **X** |  |  |  |  |
| *Lepadella patella* (Müller, 1773) | **X** | **X** |  |  |  | **X** |
| *Lepadella sp.* | **X** | **X** | **X** |  |  |  |
| *Macrochaetus* cf. *altamirai* f. *braziliensis* Koste, 1972 |  |  |  |  |  | **X** |
| *Macrochaetus* cf. *longipes* Myers, 1934 |  |  | **X** | **X** |  |  |
| *Macrochaetus* cf. *sericus* (Thorpe, 1893) |  | **X** |  |  |  | **X** |
| *Macrochaetus* spp. | **X** | **X** | **X** | **X** | **X** |  |
| *Monommata* cf. *arndti* Remane, 1933 |  |  |  |  | **X** |  |
| *Monommata* sp. |  |  |  |  |  | **X** |
| *Notomata* sp. | **X** |  |  |  |  |  |
| *Platyias quadricornis* (Ehrenberg, 1832) | **X** |  |  | **X** |  | **X** |
| *Platyias* sp. | **X** |  |  |  |  |  |
| *Plationus patulus* (Müller, 1786) | **X** | **X** |  |  |  | **X** |
| *Polyarthra* sp. | **X** | **X** | **X** | **X** | **X** | **X** |
| *Ptygura* sp. | **X** | **X** | **X** | **X** | **X** | **X** |
| *Rotaria* sp. | **X** | **X** |  |  |  |  |
| *Scaridium longicaudum* (Muller, 1786) |  | **X** |  |  |  |  |
| *Sinantherina semibullata* (Thorpe, 1889) | **X** | **X** | **X** | **X** | **X** | **X** |
| *Sinantherina spinosa* (Thorpe, 1893) |  |  |  |  | **X** |  |
| *Sinantherina* sp. | **X** | **X** | **X** |  | **X** | **X** |
| *Synchaeta* sp | **X** | **X** |  | **X** | **X** | **X** |
| *Synchaeta* cf. *stylata* Wierzejski, 1893 |  | **X** | **X** | **X** | **X** |  |
| *Testudinella* *patina* f. *trilobata* (Shephard, 1892) |  |  |  |  |  | **X** |
| *Testudinella* sp. |  |  |  |  | **X** |  |
| *Trichocerca bicristata* (Gosse, 1887) |  | **X** | **X** | **X** |  |  |
| *Trichocerca capucina* (Wierzejski & Zacharias, 1893) |  |  |  |  |  | **X** |
| *Trichocerca* *chattoni* (Beauchamp, 1907) |  | **X** |  | **X** | **X** | **X** |
| *Trichocerca cylindrica* (Imhof, 1891) | **X** | **X** | **X** | **X** | **X** | **X** |
| *Trichocerca insignis* (Herrick, 1885) |  |  |  | **X** |  |  |
| *Trichocerca pusilla* (Jennings, 1903) |  | **X** | **X** | **X** | **X** | **X** |
| *Trichocerca similis* (Wierzejski, 1893) |  | **X** | **X** | **X** |  |  |
| *Trichocerca stylata* (Gosse, 1851) |  | **X** | **X** | **X** |  |  |
| *Trichocerca* sp. | **X** | **X** | **X** | **X** | **X** | **X** |
| *Trichotria tetractis* (Ehrenberg, 1830) | **X** | **X** |  |  | **X** | **X** |
| Bdelloidea | **X** | **X** | **X** | **X** | **X** | **X** |

| **Zooplankton taxa (cont.)** | **L1** | **L2** | **L3** | **L4** | **L5** | **L6** |
| --- | --- | --- | --- | --- | --- | --- |
| **Cladocera** |  |  |  |  |  |  |
| *Acroperus tupinamba* Sniev & Elmoor-Loureiro, 2010 | **X** |  |  |  |  |  |
| *Ovalona glabra* (Sars, 1901) | **X** | **X** |  |  |  |  |
| *Alona guttata* Sars, 1862 | **X** | **X** | **X** |  |  |  |
| *Alona* sp. | **X** |  |  |  | **X** |  |
| *Bosmina hagmanni* Stingelin, 1904 | **X** | **X** | **X** | **X** | **X** | **X** |
| *Bosmina longirostris* (Muller, 1785) | **X** | **X** | **X** | **X** | **X** | **X** |
| *Bosmina* sp. | **X** | **X** | **X** | **X** | **X** | **X** |
| *Bosminopsis deitersi* Richard, 1895 | **X** |  |  |  |  |  |
| *Ceriodaphnia cornuta* Sars, 1886 | **X** | **X** | **X** | **X** | **X** | **X** |
| *Ceriodaphnia paradoxa* Spland 1926 | **X** | **X** | **X** | **X** | **X** | **X** |
| *Ceriodaphnia richardi* Sars, 1901 |  | **X** |  | **X** |  |  |
| *Ceriodaphnia silvestrii* Daday,1902 | **X** | **X** | **X** | **X** | **X** | **X** |
| *Ceriodaphnia* sp. |  | **X** |  |  |  |  |
| *Chydorus* sp. | **X** |  |  |  |  | **X** |
| *Daphnia gessneri* Herbst, 1967 | **X** | **X** | **X** | **X** | **X** | **X** |
| *Diaphanosoma birgei* Korineck, 1981 | **X** | **X** | **X** | **X** | **X** | **X** |
| *Diaphanosoma fluviatile* Hansen, 1899 | **X** |  | **X** | **X** | **X** | **X** |
| *Diaphanosoma spinulosum* Herbst, 1967 |  | **X** |  |  |  |  |
| *Diaphanosoma* sp. | **X** | **X** | **X** | **X** | **X** | **X** |
| *Disparalona* sp. | **X** | **X** |  |  |  |  |
| *Ilyocryptus spinifer* Herrick, 1882 | **X** | **X** |  | **X** | **X** | **X** |
| *Macrothrix laticornis*  Jurine, 1820 | **X** |  |  |  |  |  |
| *Macrothrix paulensis* (Sars,1901) |  |  |  |  | **X** |  |
| *Macrothrix spinosa* King, 1853 |  |  |  |  | **X** |  |
| *Moina minuta* Hansen, 1899 | **X** |  | **X** |  | **X** | **X** |
| *Moina* sp. | **X** | **X** | **X** |  |  | **X** |
| *Scapholeberis armata* (Herrick, 1882) | **X** | **X** | **X** | **X** | **X** | **X** |
| *Scapholeberis* sp. |  |  | **X** |  |  |  |
| Chydoridae | **X** | **X** |  |  |  |  |
