# Supplementary material for: Predicting temporal variation in zooplankton beta diversity is challenging
Source: PLoS One. 2017 Nov 2;12(11):e0187499. doi: 10.1371/journal.pone.0187499 (PMC5667886; doi:10.1371/journal.pone.0187499)
Supplement: S4 Table — dC Env = Environmental heterogeneity. (DOCX) [file pone.0187499.s004.docx]

**S4 Table. Temporal autocorrelation analysis of the explanatory variables used in this study.** dC Env = Environmental heterogeneity.

| Variable | Horizon | Autocorrelation | *Q* Box & Ljung | *P* |
| --- | --- | --- | --- | --- |
| Water Level | 1 | 0.86 | 48.15 | 0.000 |
|  | 2 | 0.56 | 68.82 | 0.000 |
|  | 3 | 0.21 | 71.74 | 0.000 |
|  | 4 | -0.11 | 72.56 | 0.000 |
|  | 5 | -0.33 | 80.14 | 0.000 |
|  | 6 | -0.44 | 93.63 | 0.000 |
|  | 7 | -0.43 | 106.83 | 0.000 |
|  | 8 | -0.31 | 114.01 | 0.000 |
|  | 9 | -0.13 | 115.21 | 0.000 |
|  | 10 | 0.07 | 115.56 | 0.000 |
|  | 11 | 0.21 | 118.87 | 0.000 |
|  | 12 | 0.24 | 123.55 | 0.000 |
|  |  |  |  |  |
| dC Env | 1 | 0.02 | 0.03 | 0.852 |
|  | 2 | 0.14 | 1.37 | 0.504 |
|  | 3 | 0.02 | 1.38 | 0.709 |
|  | 4 | -0.09 | 1.89 | 0.755 |
|  | 5 | -0.12 | 2.95 | 0.708 |
|  | 6 | -0.12 | 4.01 | 0.675 |
|  | 7 | 0.01 | 4.02 | 0.778 |
|  | 8 | 0.01 | 4.03 | 0.854 |
|  | 9 | 0.20 | 7.07 | 0.630 |
|  | 10 | -0.06 | 7.34 | 0.693 |
|  | 11 | -0.06 | 7.63 | 0.746 |
|  | 12 | -0.18 | 10.17 | 0.601 |
|  |  |  |  |  |
| Chlorophyll-a | 1 | 0.6 | 23.73 | 0.000 |
|  | 2 | 0.3 | 29.57 | 0.000 |
|  | 3 | 0.15 | 31.18 | 0.000 |
|  | 4 | 0.09 | 31.75 | 0.000 |
|  | 5 | -0.12 | 32.77 | 0.000 |
|  | 6 | -0.22 | 36.15 | 0.000 |
|  | 7 | -0.12 | 37.12 | 0.000 |
|  | 8 | -0.11 | 38.07 | 0.000 |
|  | 9 | -0.16 | 40.05 | 0.000 |
|  | 10 | -0.13 | 41.33 | 0.000 |
|  | 11 | -0.06 | 41.58 | 0.000 |
|  | 12 | -0.02 | 41.63 | 0.000 |
