# Supplementary material for: Predicting temporal variation in zooplankton beta diversity is challenging
Source: PLoS One. 2017 Nov 2;12(11):e0187499. doi: 10.1371/journal.pone.0187499 (PMC5667886; doi:10.1371/journal.pone.0187499)
Supplement: S5 Table — dBC = average distance to group centroid; βNES = Nestedness component. Models were run for each zooplankton group separately. (DOCX) [file pone.0187499.s005.docx]

**S5 Table. Akaike Information Criterion (AIC_c_), delta AIC and Akaike weights for models with different autocorrelation structures (assuming no autocorrelation (OLS), ARMA(1,0), ARMA(2,0) and Compound Symmetry).** dBC = average distance to group centroid; βNES= Nestedness component. Models were run for each zooplankton group separately.

| **Testate Amoebae** | | | | |
| --- | --- | --- | --- | --- |
|  |  |  |  |  |
| **Beta diversity**  **measure** | **Models** | **AICc** | **delta**  **AICc** | **Weight** |
| dBC | OLS | -48.0 | 0.00 | 0.53 |
|  | ARMA(1,0) | -46.1 | 1.90 | 1.90 |
|  | Compound Symmetry | -45.4 | 2.50 | 0.53 |
|  | ARMA(2,0) | -44.9 | 3.10 | 3.10 |
|  |  |  |  |  |
| βNes | OLS | 44.4 | 0.00 | 0.59 |
|  | ARMA(1,0) | 46.9 | 2.50 | 0.17 |
|  | Compound Symmetry | 47.0 | 2.60 | 0.16 |
|  | ARMA(2,0) | 48.4 | 3.90 | 0.08 |
|  |  |  |  |  |
| **Rotifera** | | | | |
|  |  |  |  |  |
| **Beta diversity**  **measure** | **Models** | **AICc** | **delta**  **AICc** | **Weight** |
| dBC | ARMA(1,0) | -113.8 | 0.00 | 0.74 |
|  | ARMA(2,0) | -111.3 | 2.50 | 0.21 |
|  | OLS | -107.9 | 5.90 | 0.04 |
|  | Compound Symmetry | -105.4 | 8.40 | 0.01 |
|  |  |  |  |  |
| βNes | ARMA(2,0) | -99.86 | 0.00 | 0.78 |
|  | ARMA(1,0) | -91.54 | 3.10 | 0.17 |
|  | OLS | -94.09 | 5.80 | 0.04 |
|  | Compound Symmetry | -96.79 | 8.30 | 0.01 |
|  |  |  |  |  |
| **Cladocera** | | | | |
|  |  |  |  |  |
| **Beta diversity**  **measure** | **Models** | **AICc** | **delta**  **AICc** | **Weight** |
| dBC | ARMA(1,0) | -72.79 | 0.00 | 0.73 |
|  | ARMA(2,0) | -70.15 | 2.60 | 0.19 |
|  | OLS | -67.80 | 5.00 | 0.06 |
|  | Compound Symmetry | -65.25 | 7.50 | 0.02 |
|  |  |  |  |  |
| βNes | OLS | -13.57 | 0.00 | 0.55 |
|  | ARMA(1,0) | -11.32 | 2.20 | 0.18 |
|  | Compound Symmetry | -11.02 | 2.50 | 0.15 |
|  | ARMA(2,0) | -10.46 | 3.10 | 0.12 |
