# Supplementary material for: Predicting temporal variation in zooplankton beta diversity is challenging
Source: PLoS One. 2017 Nov 2;12(11):e0187499. doi: 10.1371/journal.pone.0187499 (PMC5667886; doi:10.1371/journal.pone.0187499)
Supplement: S6 Table — SE = Standard Error; dC Env = Environmental heterogeneity; dBC = average distance to group centroid; βNES = Nestedness component. (DOCX) [file pone.0187499.s006.docx]

**S6 Table. Best selected models for each zooplankton group beta diversity (dBC and βNes) at Ribeirão das Lajes Reservoir, Brazil (see Table S5).** *SE* =Standard Error; dC Env = Environmental heterogeneity; dBC= average distance to group centroid; βNES = Nestedness component.

| **Testate Amoebae** | | | | | | |
| --- | --- | --- | --- | --- | --- | --- |
|  |  |  |  |  |  |  |
| **Beta diversity**  **measure** | **Models** | **Variables** | **Coeff.** | ***SE*** | ***t*** | ***P*** |
| dBC | OLS | (Intercept) | 3.572 | 3.752 | 0.952 | 0.345 |
|  |  | Time | -0.001 | 0.001 | -1.143 | 0.258 |
|  |  | Chlorophyll-a | -0.021 | 0.015 | -1.422 | 0.161 |
|  |  | dC Env | -0.001 | 0.027 | -0.039 | 0.969 |
|  |  | Water level | -0.008 | 0.009 | -0.836 | 0.406 |
|  |  |  |  |  |  |  |
|  | ARMA(1,0) | (Intercept) | 3.518 | 4.144 | 0.849 | 0.400 |
|  |  | Time | -0.001 | 0.001 | -1.023 | 0.311 |
|  |  | Chlorophyll-a | -0.020 | 0.016 | -1.250 | 0.217 |
|  |  | dC Env | 0.005 | 0.026 | 0.176 | 0.861 |
|  |  | Water level | -0.008 | 0.010 | -0.747 | 0.458 |
|  |  |  |  |  |  |  |
| βNes | OLS | (Intercept) | 12.345 | 8.526 | 1.448 | 0.153 |
|  |  | Time | 0.000 | 0.002 | -0.024 | 0.981 |
|  |  | Chlorophyll-a | 0.041 | 0.033 | 1.241 | 0.220 |
|  |  | dC Env | -0.014 | 0.061 | -0.229 | 0.819 |
|  |  | Water level | -0.029 | 0.021 | -1.416 | 0.162 |
|  |  |  |  |  |  |  |
|  | ARMA(1,0) | (Intercept) | 12.363 | 8.791 | 1.406 | 0.165 |
|  |  | Time | 0.000 | 0.002 | -0.032 | 0.975 |
|  |  | Chlorophyll-a | 0.040 | 0.034 | 1.186 | 0.241 |
|  |  | dC Env | -0.013 | 0.061 | -0.220 | 0.827 |
|  |  | Water level | -0.029 | 0.021 | -1.375 | 0.175 |
|  |  |  |  |  |  |  |
| **Rotifera** | | | | | | |
|  |  |  |  |  |  |  |
| **Beta diversity**  **measure** | **Models** | **Variables** | **Coeff.** | ***SE*** | ***t*** | ***P*** |
| dBC | ARMA(1,0) | (Intercept) | 4.179 | 3.008 | 1.389 | 0.170 |
|  |  | Time | **-0.002** | **0.001** | **-2.673** | **0.010** |
|  |  | Chlorophyll-a | 0.000 | 0.010 | -0.034 | 0.973 |
|  |  | dC Env | 0.011 | 0.014 | 0.834 | 0.408 |
|  |  | Water level | -0.009 | 0.007 | -1.285 | 0.204 |
|  |  |  |  |  |  |  |
|  | ARMA(2,0) | (Intercept) | 4.194 | 2.977 | 1.409 | 0.164 |
|  |  | Time | **-0.002** | **0.001** | **-2.788** | **0.007** |
|  |  | Chlorophyll-a | 0.000 | 0.010 | 0.022 | 0.983 |
|  |  | dC Env | 0.011 | 0.014 | 0.825 | 0.413 |
|  |  | Water level | -0.009 | 0.007 | -1.304 | 0.197 |
|  |  |  |  |  |  |  |
| βNes | ARMA(2,0) | (Intercept) | -0.050 | 3.233 | -0.016 | 0.988 |
|  |  | Time | 0.000 | 0.001 | 0.053 | 0.958 |
|  |  | Chlorophyll-a | 0.011 | 0.011 | 1.072 | 0.288 |

| **Table S6 (cont.)** |  |  |  |  |  |  |
| --- | --- | --- | --- | --- | --- | --- |
|  |  | dC Env | 0.009 | 0.016 | 0.587 | 0.559 |
|  |  | Water level | 0.000 | 0.008 | 0.058 | 0.058 |
|  |  |  |  |  |  |  |
|  | ARMA(1,0) | (Intercept) | 2.003 | 3.235 | 0.619 | 0.538 |
|  |  | Time | 0.000 | 0.001 | 0.195 | 0.846 |
|  |  | Chlorophyll-a | 0.006 | 0.011 | 0.576 | 0.567 |
|  |  | dC Env | -0.003 | 0.016 | -0.189 | 0.851 |
|  |  | Water level | -0.004 | 0.008 | -0.569 | 0.572 |
|  |  |  |  |  |  |  |
| **Cladocera** | | | | | | |
|  |  |  |  |  |  |  |
| **Beta diversity**  **measure** | **Models** | **Variables** | **Coeff.** | ***SE*** | ***t*** | ***P*** |
| dBC | ARMA(1,0) | (Intercept) | 6.683 | 4.351 | 1.536 | 0.130 |
|  |  | Time | **-0.002** | **0.001** | **-2.154** | **0.036** |
|  |  | Chlorophyll-a | -0.016 | 0.014 | -1.094 | 0.279 |
|  |  | dC Env | -0.012 | 0.020 | -0.623 | 0.536 |
|  |  | Water level | -0.015 | 0.011 | -1.440 | 0.155 |
|  |  |  |  |  |  |  |
|  | ARMA(2,0) | (Intercept) | 6.683 | 4.351 | 1.536 | 0.130 |
|  |  | Time | **-0.002** | **0.001** | **-2.154** | **0.036** |
|  |  | Chlorophyll-a | -0.016 | 0.014 | -1.094 | 0.279 |
|  |  | dC Env | -0.012 | 0.020 | -0.624 | 0.535 |
|  |  | Water level | -0.015 | 0.011 | -1.441 | 0.155 |
|  |  |  |  |  |  |  |
| βNes | OLS | (Intercept) | -2.914 | 5.075 | -0.574 | 0.568 |
|  |  | Time | 0.001 | 0.001 | 1.079 | 0.285 |
|  |  | Chlorophyll-a | 0.001 | 0.020 | 0.062 | 0.951 |
|  |  | dC Env | 0.050 | 0.036 | 1.387 | 0.171 |
|  |  | Water level | 0.008 | 0.012 | 0.612 | 0.543 |
|  |  |  |  |  |  |  |
|  | ARMA(1,0) | (Intercept) | -2.760 | 4.713 | -0.586 | 0.560 |
|  |  | Time | 0.001 | 0.001 | 1.096 | 0.278 |
|  |  | Chlorophyll-a | 0.000 | 0.019 | -0.009 | 0.993 |
|  |  | dC Env | 0.058 | 0.036 | 1.618 | 0.111 |
|  |  | Water level | 0.007 | 0.011 | 0.625 | 0.535 |
